# Estimating the fraction of falsely detected spikes in high density microelectrode array recordings based on correlations

**DOI:** 10.1186/1471-2202-14-S1-P25

**Published:** 2013-07-08

**Authors:** Jens-Oliver Muthmann, Hayder Amin, Alessandro Maccione, Evelyne Sernagor, Luca Berdondini, Matthias H Hennig, Upinder S Bhalla

**Affiliations:** 1 National Centre for Biological Sciences, Tata Institute of Fundamental Research, Bangalore, 560065, India; 2 Institute for Adaptive and Neural Computation, University of Edinburgh, Edinburgh, EH8 9AB, UK; 3 Department of Neuroscience and Brain Technologies, Istituto Italiano di Tecnologia, Genova, Italy; 4 Institute of Neuroscience, Newcastle University Medical School, Newcastle upon Tyne, UK

## 

High-density microelectrode arrays (MEA) can measure neuronal activity in potentially thousands of units with a high spatial resolution [1]. However due to the small size of the preamplifers, noise artifacts can affect spike detection. Additionally, the MEA chip itself is not perfectly homogeneous and the electrical coupling between the electrodes and a neuron may be weak. Therefore, the characteristics of neuronal spikes and noise are inherently different in each recording channel, such that estimating an average performance of the spike detection would not be representative for individual recording channels. As we aim to observe slow changes in single neuron activity, it is crucial to know how much a change in electrical coupling could potentially affect the number of detected spikes.

Here we estimate the quality of spike detection using correlations as an indicator to distinguish between neuronal activity and noise. First we use a threshold-based detection of putative spikes, with a deliberately low threshold to lower the number of undetected spikes. We estimate pairwise correlations between spike trains based on spike ranks rather than time in order to reduce the effects of nonstationarities in the recordings. We then assume that the frequency of spontaneous firing in the neurons is small compared to the evoked firing and check for each detected spike whether we can find correlated units which are active within a short interval around its spike time. For each unit, we compare the relative frequency of such correlated spikes to the frequency that would be obtained by doing the same analysis with a Poisson spike train.

**Figure 1:**
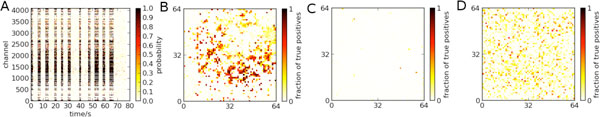
**Correlation-based estimate of the fraction of spikes corresponding to neuronal activity for a low detection threshold**. Hippocampal culture, 15 min recording. **A**: Rasterplot of the activity. Dark colors correspond to highly correlated spikes. **B**: Fractions classified as neuronal spikes. **C**: Results after shuffling the channel identities of spikes to preserve nonstationarities and different firing rates. **D**: Result for a 30 min recording of an empty MEA.

This yields a per unit estimate of the fraction of putatively falsely detected spikes and we validate the procedure using shuffled data and recordings of an empty MEA (see Figure 1.). To investigate the dependency of this method on a specific network dynamics, we apply it to a few sample recordings of a hippocampal culture and a perinatal retina. It is possible to exclude spikes with a low amplitude after doing this analysis and this decreases the fraction of falsely detected spikes, but for the hippocampal cultures this leads to a loss of detected spikes in many units.
